# Reference Data for a Treadmill-Based Peak Oxygen Uptake (VO_2peak_) in Older Adults

**DOI:** 10.3390/biology14020128

**Published:** 2025-01-26

**Authors:** Peter Sagat

**Affiliations:** GSD/Health and Physical Education Department, Sport Sciences and Diagnostics Research Group, Prince Sultan University, Riyadh 11586, Saudi Arabia; petersagatdr@gmail.com

**Keywords:** VO_2peak_, geriatrics, sex differences, age differences, cardiorespiratory fitness, standards

## Abstract

**Background:** Although the level of cardiorespiratory fitness (CRF) in older adults has been well documented, little evidence has been provided regarding sex- and age-specific reference standards. Therefore, the main purpose of the study was to examine objectively measured CRF in older adults based on sex and age. **Methods:** This observational study included 450 men (N = 170) and women (N = 280) aged ≥60 years with mean ± standard deviation (SD) for age (67.4 ± 5.4), height (165.7 ± 8.6 cm), weight (75.1 ± 13.3 kg) and body mass index (BMI, 27.1 ± 3.9 kg/m^2^). Participants performed an incremental exercise ramp test until exhaustion on a treadmill, and the outcome of CRF was presented as peak oxygen uptake (VO_2peak_) in milliliters per kilogram of body mass per minute (ml/kg/min). **Results:** Relative VO_2peak_ values were 37.3 ± 7.1, 30.3 ± 6.6, 24.8 ± 5.4 and 21.7 ± 5.8 mL/kg/min for men aged 60–64 years, 65–69 years, 70–74 years and ≥75 years. For the same age groups in women, values were 30.5 ± 4.8, 27.1 ± 5.4, 22.4 ± 3.4 and 18.9 ± 4.7 mL/kg/min. Age-related declines were observed in both men and women (*p* < 0.001). In a sex–age interaction term, significant main effects occurred, where men in every age group exhibited higher VO_2peak_ values, compared to women in the same age groups. **Conclusions:** This study shows that CRF needs to be assessed by considering sex and age as significant predictors. Thus, sex- and age-specific reference findings may serve as an avenue to monitor, track and compare between older adults of different nationalities.

## Simple Summary

The study examined peak oxygen uptake (VO_2peak_) using reference data for older adults aged ≥ 60 years. Four-hundred and fifty men and women completed procedures on a treadmill. Participants were categorized into men and women in the four age groups (60–64 years, 65–69 years, 70–74 years and ≥75 years). When analyzing sex- and age-related differences, findings suggested that men (sex) and younger men and women (age) exhibited higher values in VO_2peak_, compared to women, and older men and women, respectively. When the main interaction effects between sex and age were observed, men in every age group performed better in VO_2peak_, compared to women in the same age groups. This study confirms that sex and age play an important role in measuring cardiorespiratory performance in older adults. Thus, these simple parameters should be taken into account when establishing norms in future research.

## 1. Introduction

Cardiorespiratory fitness (CRF), along with muscular fitness, flexibility and body composition, represents a physical characteristic aiming to efficiently deliver and use oxygen for the musculoskeletal system and physical activity (PA) [1]. A low level of CRF has been consistently associated with adverse health outcomes, including cardiovascular [2,3], metabolic [4] and mental diseases [5] and premature mortality [6]. In older adults, poor CRF has been even more pronounced and often accompanied by a decrease in skeletal muscle mass and function [7]. Indeed, the prevalence of older adults is rapidly growing [8], and estimates suggest that by 2050, the population of older adults will reach or even surpass two billion, accounting for 22% of the global population [9]. Although life expectancy is increasing [10] and the benefits of high CRF have been well established [11], it has not been part of the existing cardiovascular-scale models [12,13,14], and it has remained underutilized in clinical practice [15]. Thus, its applicability and prognostic utility should be incorporated in prevention and rehabilitation medicine [15,16].

The outcome measure of CRF is often defined by the peak oxygen uptake (VO_2peak_) obtained from incremental exercise testing [16]. The nature of testing is based on exposing an individual to an incremental work rate leading to a volitional work rate. However, the final score must be controlled for sex and age, because previous research has highlighted the importance of putting CRF into the sex- and age- specific reference values [17]. It has been reported that age alone accounts for 30–40% of variation in CRF [18], and age-related decline of CRF is around 10–15% in older adults per decade [19]. On the other hand, men generally outperform women, which is especially noticeable after the age of 60 years [16]. Thus, CRF of an individual should be described and compared to other peers in terms of sex and age, to optimize risk assessment and meeting the recommended guidelines [20].

To date, several studies have examined sex- and age-specific reference values of CRF in older adults [16,17,18,20,21,22,23,24,25,26,27]. However, most of these studies have examined reference data for VO_2peak_ across a wide age range with the largest sample being presented for general population [16,17,18,20,21,22,23,24,25,26,28], while more thorough insights for older adults have been lacking [27]. A study by Stensvold et al. [27] has shown a significant sex and age differences, where men exhibit higher VO_2peak_ values, compared to women (31.3 ± 6.7 mL/kg/min vs. 26.2 ± 5.0 mL/kg/min) and older men and women are outperformed by their younger counterparts. Similar values have been confirmed in older adults from the United States [18,20] and Norway [26]. Although CRF has been confirmed as an important determinant of present and future health [29], the existing sex- and age-specific data in older adults are somewhat scarce. Examining such data worldwide would give the possibility to compare, monitor and track cardiopulmonary outcome measures in different sex, age and ethnic groups. Moreover, establishing reference values in clinical practice is always challenging, because an individual must be motivated through the whole testing procedure, which may influence true results. Also, previous evidence has highlighted the importance of cardiopulmonary exercise testing in relation to muscle strength and physical performance, where a higher VO_2peak_ is constantly associated with higher leg and grip strength [30]. Such findings may have even more clinical utility related to locomotor functionality status and engaging in activities of daily living than mortality or disease risks [31,32]. In that way, information about cardiopulmonary function broadens its clinical impact on other health outcomes, as it may also provide important details regarding muscular strength status and performance in healthy older adults. Given the importance of functional performance declining with age, a systematic screening of cardiopulmonary exercise testing would be useful in distinguishing between normal and abnormal responses, identifying factors (in this case sex and age) related to cardiopulmonary limitations, and examining prognostic ability and evaluating the effectiveness of therapeutic interventions [33].

Therefore, the main purpose of the study was to provide sex- and age-specific reference values for CRF in older adults aged ≥60 years and to examine whether sex, age and the interaction between sex and age had a role in VO_2peak_ distribution. Based on previous findings [18,20,26,27], it was hypothesized that sex-related analysis would reveal that men exhibited higher CRF values compared to women; age-related analysis would confirm that younger men and women performed better than their older counterparts; and sex–age interaction would show that men in a certain age group outperformed women in the same age group, respectively.

## 2. Materials and Methods

### 2.1. Study Participants

In this observational study, 520 older adults aged ≥60 years were recruited who were annually examined for CRF in four high-quality clinics from May to September 2024. All clinics and their physiology laboratories used the same procedures to examine and test CRF. Since the aim was to observe age and sex reference data for CRF in apparently healthy individuals, the inclusion criteria included predominantly sedentary older adults aged ≥60 years without major non-communicable diseases (cardiovascular, metabolic or mental), which could prevent them from completing the testing procedures, but who could complete daily activities without exhaustion or independently of others; individuals who were in normal or slightly overweight body mass index (BMI) categories (range between 23.0 and 28.0 kg/m^2^, mean value for our sample = 27.1 kg/m^2^), where previous studies have shown that the majority of older adults are categorized as being ‘overweight’ [34]; women who were not in the menopause status and both men and women who were free of medication which could act contradictory to testing outcomes; and being able to perform a treadmill-based procedure. Of 520 older adults, 58 failed to perform an exercise until the end, and 12 did not want to participate in the study. Thus, our final sample included 450 (61.9% women) aged ≥60 years of age. Since the main purpose of the study was to identify specific differences between age groups, a post hoc analysis to obtain the required statistical power of the findings using a G*power analysis software (v.3.1.9.7) calculator was used [35]. To achieve a moderate effects size of f = 0.25, α level of <0.05 and total sample size of N = 450, with the number of groups being N = 8 (men and women in four age-related groups), the achieved power was 1 − β = 0.97. Before the study had begun, all participants were instructed about the general purpose and expected outcomes. All procedures were anonymous and voluntary, meaning that each participant had the right to withdraw at any time. Research involving human subjects complied with all relevant national regulations and institutional policies, is in accordance with the tenets of the Helsinki Declaration (as revised in 2013) and has been approved by the Ethical Committee of the local Home for older adults (Ethical code: 2024/4/18).

### 2.2. VO_2peak_

CRF was tested on a treadmill, using a ramp protocol to determine VO_2peak_. Based on previous studies, VO_2peak_ was defined as the mean of VO_2_ in the last 10 s of the exercise test [16] and presented as a relative value in ml/kg/min. Before testing, each participant had a warm-up period of 8–10 min initiated from the inclination of a treadmill, while wearing the COSMED K4b^2^ (Rome, Italy) mask. A modified Bruce protocol with individualized speed increases (1.5 m/s) and inclination increases (15%) for every 3 min until exhaustion was used. Every clinic was instructed to do the testing under the same conditions, at room temperature (22–24 °C) and with all tests being supervised by certified physiologists. Before the testing, physiologists went through a standardized procedure of examining VO_2peak_. Also, all participants were told not to ingest caffeine for 12 h or eat within 3 h prior to exercise testing, and to come to the test in clothes suitable for performing the exercise.

### 2.3. Clinical Parameters

Height (a portable stadiometer) and weight (a digital scale) were objectively measured, and BMI was calculated using the following formula: weight (kg)/height (m^2^). Each participant was instructed to stand still in an upright position to measure height, after which a calibrated digital scale was used to examine weight. Waist circumference (WC) was measured with anthropometric tape placed horizontally midway between the lower rib margin and the iliac crest at the end of normal expiration. The waist-to-height ratio (WHtR) was calculated by dividing WC with height. To assess systolic (SBP) and diastolic (DBP) blood pressure, a standard mercury sphygmomanometer blood pressure cuff on the right mid-arm at the same level as the heart was used, having three trials in a sitting position after a three min rest period between each trial, after which the average of three measurements was used in the analyses. SBP was evaluated by the first blood flow sound re-established after the pressure was applied, and DBP was recorded as the final sound of decompressing the blood pressure cuff. Although the data showed that the mean SBP was above 140, which would indicate hypertension in older adults [36], all participants had undergone a complete physical examination and were approved to continue with testing. Fat mass percentage (%) was measured using bioelectrical impedance analysis (Omron BF500 Body Composition Monitor, Omron Medizintechnik, Hamburg, Germany). Before an individual stood on metal pads, sex, age and height were entered into the device, to examine the estimated body composition parameters. After the initial protocol, each participant stood on metal pads of the scale and grasped a pair of electrodes with arms in an extension position for 10 sec, after which the data regarding fat mass % were generated. Heart rate at rest (HR _resting_) and at peak level (HR _max_) were measured automatically with a Philips IntelliVue MP50 (Philips Medizin Systeme, Boeblingen, Germany) at the beginning and at the end of the testing procedure, as described in previous studies [27]. Blood samples were collected in the morning hours in a resting seated position after a 12 h overnight fast. Blood samples were drawn from the forearm using a vacutainer blood collection tube with a needle. Laboratory measurement of fasting blood glucose and total cholesterol were extracted using the Cholestech LDX^®^ analyzer (Abbott™ 10959, Des Plaines, IL, USA).

### 2.4. Data Analysis

Data statistics are presented as mean and standard deviation (SD), based on the normality Kolmogorov–Smirnov test. The analysis of variance (ANOVA) with sex (men vs. women) and age (60–64 years, 65–69 years, 70–74 years and ≥75 years) and their interaction (sex–age) was used to assess the main effects for VO_2peak_. All analyses were performed in Statistical Packages for Social Sciences version 26 (SPSS Inc., Chicago, IL, USA), and the significance was set at *p* < 0.05 (two-sided).

## 3. Results

Basic descriptive data of the study participants can be found in Table 1. Older men were taller and heavier and had higher WC, SBP _resting_ and glucose values compared to women (*p* ≤ 0.05). Women exhibited higher values in fat mass, HR _resting_ and total cholesterol (*p* ≤ 0.05). Interestingly, no significant differences in BMI, WHR, DBP _resting_ and HR _max_ were observed (*p* > 0.05). Of note, the prevalence of normal weight (<25 kg/m^2^) and overweight (25.0–29.9 kg/m^2^) in this sample was 54.0% and 46%, respectively. The prevalence of older adults with an SBP over 120 mmHg and 140 mmHg was 37.3% and 27.4%.

**Figure 1:**
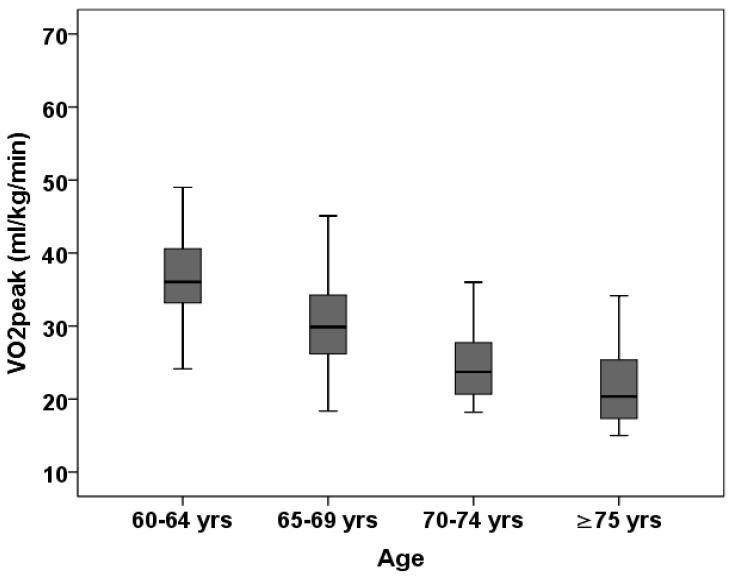
Box plot of relative VO_2peak_ by age group in men (N = 170); data are presented as median and interquartile range (25th–75th percentile).

Figure 1 shows age-specific mean values of VO_2peak_ in men. For men aged 60–64 years, 65–69 years, 70–74 years and ≥75 years, mean VO_2peak_ values were 37.3 ± 7.1 mL/kg/min, 30.3 ± 6.6 mL/kg/min, 24.8 ± 5.4 mL/kg/min and 21.7 ± 5.8 mL/kg/min. A Bonferroni post hoc analysis showed that men in the youngest age group had significantly higher values in VO_2peak_, compared to men in other age groups. Non-significant differences were only found between 70–74 years and ≥75 years age categories (mean diff. = −3.1 mL/kg/min, *p* = 0.921).

**Figure 2:**
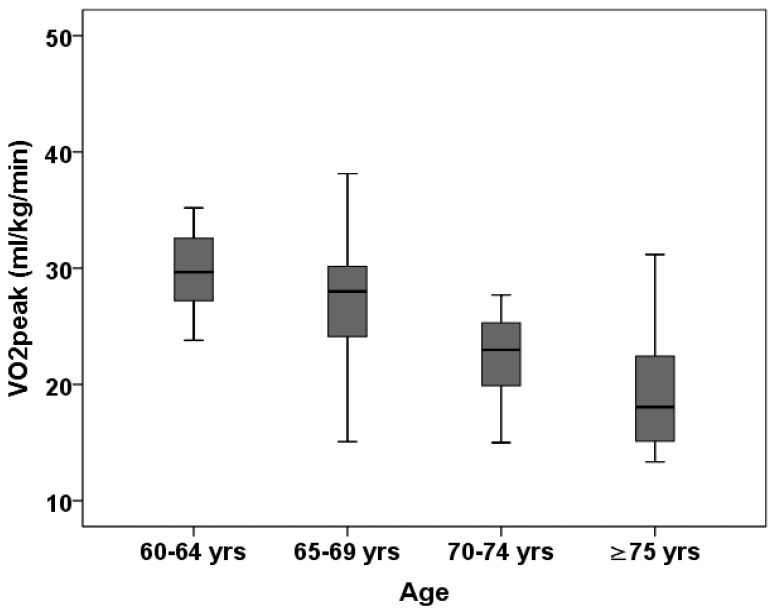
Box plot of relative VO_2peak_ by age group in women (N = 280); data are presented as median and interquartile range (25th–75th percentile).

Figure 2 shows age-specific mean values of VO_2peak_ in women. A similar age-related decline was observed in women as for men. VO_2peak_ mean values for women aged 60–64 years, 65–69 years, 70–74 years and ≥75 years were 30.5 ± 4.8 mL/kg/min, 27.1 ± 5.4 mL/kg/min, 22.4 ± 3.4 mL/kg/min and 18.9 ± 4.7 mL/kg/min. Similar to men, a Bonferroni post hoc analysis also showed that women in the youngest age group had significantly higher values in VO_2peak_, compared to women in other age groups. Non-significant differences between women aged 70–74 years and ≥75 years (mean diff. = −3.6 mL/kg/min, *p* = 0.098) were observed.

Table 2 presents reference data for VO_2peak_ by sex and age. Compared to women, men exhibited higher values in mean VO_2peak_ (28.5 ± 7.6 mL/kg/min vs. 24.7 ± 6.5 mL/kg/min, *F*_3,449_ = 22.777, mean diff. = 3.8 mL/kg/min, *p* < 0.001). A significant age-related decrease in mean VO_2peak_ values for men and women aged 60–64 years (33.9 ± 8.4 mL/kg/min), 65–69 years (28.8 ± 7.1 mL/kg/min), 70–74 years (23.6 ± 5.9 mL/kg/min) and ≥75 years (20.3 ± 5.5 mL/kg/min) were observed (*F*_3,449_ = 63.671, *p* < 0.001). The interaction term between sex and age showed significant main effects (*F*_3,449_ = 4.451, *p* < 0.001), where men in all age groups performed better, compared to women in the same age groups, respectively. Specifically, men had superior values in VO_2peak_ compared to women in the 60–64 years (37.3 ± 7.1 vs. 30.5 ± 4.8 mL/kg/min), 65–69 years (30.3 ± 6.6 vs. 27.1 ± 5.4 mL/kg/min), 70–74 years (24.8 ± 5.4 vs. 22.4 ± 3.4 mL/kg/min) and ≥75 years groups (21.7 ± 5.8 vs. 18.9 ± 4.7 mL/kg/min).

## 4. Discussion

The main purpose of this study was to provide reference values for CRF in older adults aged ≥ 60 years. The main findings of the study are as follows: (i) men have higher VO_2peak_ values compared to women; (ii) an age-related decline is observed in both men and women, where younger men and women exhibit better results compared to their older counterparts; and (iii) when an interaction between sex and age is postulated, men in all age groups have higher VO_2peak_ values, compared to women in the same age groups.

Our findings that men exhibited higher VO_2peak_ values compared to women are consistent and in line with previous studies [16,18,20,26,27]. The data from the Fitness Registry and the Importance of Exercise National Database (FRIEND) [20] have shown that the means of relative VO_2peak_ measured with treadmill in men aged 60–69 years, 70–79 years and 80–89 years are 25.4 mL/kg/min, 21.2 mL/kg/min and 17.9 mL/kg/min, and for women in the same age groups, these values are 20.0 mL/kg/min, 175 mL/kg/min and 15.9 mL/kg/min, respectively. Similar observations have been obtained in a study by Rapp et al. [16], where mean values ranged between 29.0 mL/kg/min in men and 24.0 mL/kg/min in women above the age of 60 years, respectively. In one of the largest reference materials on directly measured CRF in older adults aged 70 to 77 years, men had higher VO_2peak_ compared to women (31.3 ± 6.7 mL/kg/min vs. 26.2 ± 5.0 mL/kg/min) [27]. In general, men have consistently better results in CRF compared to women. From a biological point of view, sex-related differences are often accompanied by women reaching exercise-induced arterial hypoxemia at lower intensities [37], having smaller lung capacities [38] and lower expiratory force rates, due to the smaller airway diameters and lung volumes, resulting in restricted expiratory flow [39] and a reduced diffusion capacity [40]. Also, women often tend to have lower levels of PA compared to men [16,27]. However, from a physiological perspective, men are generally more physically active and have a greater skeletal muscle mass [7]. On the other hand, these results suggested that men consumed oxygen less efficiently than women during the treadmill exercise, that is, men needed more oxygen for the same power output compared to women. The results from this study confirmed the findings from previous studies [27], where men tended to exhibit better cardiopulmonary function compared to women. The mechanisms are related to sex differences in CRF, pointing out that reference standards should be sex-specific, to generalize the findings to older adults from different nationalities.

It was observed that younger men and women (60–64 years) exhibited higher VO_2peak_ values, in comparison to their older counterparts, which is also in line with previous findings in older adults [18,20]. For example, in a study by Kaminsky et al. [20], mean VO_2peak_ values were 25.4 mL/kg/min, 21.2 mL/kg/min and 17.9 mL/kg/min for men aged 60–69 years, 70–79 years and 80–89 years, and in women within the same age groups, mean values were 20.0 mL/kg/min, 17.5 mL/kg/min and 15.9 mL/kg/min, respectively. Another study exhibited somewhat higher VO_2peak_ values of 38.5 mL/kg/min and 34.1 mL/kg/min in men and 30.6 mL/kg/min and 26.5 mL/kg/min in women at ages 60–69 years and ≥70 years [18]. In both studies, the average % of change per decade was between 11 and 16% [18,20]. In our study, the largest declines were observed between men aged 60–64 years and 65–69 years and women between 65–69 years and 70–74 years with an average overall % of decline of 16% in men and 17% in women. In general, a systematic review by Letnes et al. [19] has shown that annual age-related declines in cross-sectional studies range between 0.40 mL/kg/min for active and sedentary men and 0.35 mL/kg/min to 0.44 mL/kg/min in women, which represents approximately a 10% decline per decade. Interestingly, evidence from longitudinal studies suggests a decade decline at about 15% after 60 years of age [19]. It has been suggested that older age is often accompanied by a higher number of comorbidities, which has been associated with lower improvements in CRF and other outcomes [41,42]. Studies have shown great hormonal changes related to cardiopulmonary performance, where the level of testosterone tends to decline more rapidly after the age of 40 [43]. Such patterns directly affect the progressive reduction in skeletal muscle mass and lower relative muscle strength and endurance. However, age-related declining trends between men and women show that muscle mass decreases somewhat more between 50 and 60 years in men, while women experience a stagnation during the same age period, but more rapid decreases after the age of 60 [44]. To support these constatations, a sub-analysis from this study showed that fat mass was inversely correlated with VO_2peak_ in men (*r* = −32) but was more pronounced in women (*r* = −0.43). This would suggest that less skeletal muscle mass and more fat mass in women in accordance with age had more adverse outcomes to cardiopulmonary functional status, compared to men in the same age groups.

Finally, significant main effects in the interaction between sex and age were observed, where VO_2peak_ values in a certain age category were more pronounced in men, opposed to women, and these differences were achieved in all age groups. According to the literature and sex- and age-related differences [39,40,42], CRF should always be tested in relation to sex and age. Although an effort has been made to establish such data for CRF, certain discrepancies between the studies should be mentioned. Somewhat different values of VO_2peak_ may be explained by using age as a categorical predictor in 10-year age classes [16,18,20], while the study used more detailed 5-year age categories. Also, a few studies have conducted measurements in unhealthy participants mostly affected by cardiovascular diseases [27] with relatively small sample sizes in each age group [16,18,20,27]. Such limitations in previous studies might have led to insufficient statistical power and the impossibility of data generalization to populations of other nationalities.

Observing objectively measured CRF in both clinical and population-based settings should be a cornerstone for initially monitoring and tracking aerobic performance. By establishing sex- and age-referenced values, health- and research-related professionals would be able to screen for older adults with poor CRF and how these data change, according to other sex and groups. Thus, interventions in the most vulnerable populations should be applied to test the effectiveness and applicability [45]. Also, practical implication of the obtained data should serve as an assessment of the integrated response to cardiovascular, respiratory and musculoskeletal systems to exercise. The information related to one’s functional performance is connected to improving the diagnosis or therapeutic management in non-communicable diseases. On the other hand, cardiopulmonary exercise testing represents a gold standard for aerobic quantification in both affected and healthy individuals. Thus, the utility of undertaking such a procedure in both clinical and population-based settings should be the basis for detecting and prescribing adequate intervention programs, especially for individuals with cardiovascular and pulmonary limitations.

This study has a few limitations. Unfortunately, a cross-sectional design cannot show causal effects of sex and age on CRF. Indeed, the existing literature highlighted some changes between cross-sectional and longitudinal designs, where longitudinal, repeated observations were reliable and valid estimates on declines in CRF associated with aging [46]. The participants recruited in this study were apparently healthy older adults being tested for their annual systematic examinations, while the data were not compared with their counterparts affected by chronic diseases, like analyzed in previous studies [27]. Third, it has been highlighted that, in future studies, age should be observed as a continuous variable, because an age-related annual decline is between 10 and 15% [19], and participants within the same age group without the same chronological age may have exhibited different results. Fourth, although the findings were based on a relatively large number of participants with a satisfactory statistical power, sex- and age-related differences decreased the number of individuals within each group, which could prevent a generalizable conclusion from being made regarding similar sex and age groups of older adults. Fifth, the characteristics of the sample size were apparently healthy individuals without major diseases. Based on the obtained data, the results showed that the mean values for BMI and SBP were 27.1 kg/m^2^ and 142.6 mmHg, which were pre-defined states for future diseases. Although all participants were given permission to participate in the study, more detailed analyses regarding BMI- and hypertension-specific data should be calculated. However, a correlation analysis showed only a trivial to small relationship between VO_2peak_ and BMI (*r* = −0.38) and SBP (*r* = −0.08). Thus, these risk factors, although important for cardiopulmonary testing, did not seem to largely influence the findings. Finally, the testing relied on one’s motivation to perform as well as possible. For motivational purposes to be included in the study and to perform the testing at a maximum level, the data were not collected, and the findings should be interpreted with caution. Therefore, future longitudinal, population-based studies in healthy and unhealthy older adults controlled for a variety of risk factors are warranted.

## 5. Conclusions

In summary, this study established sex- and age-related reference values for CRF (VO_2peak_ as an outcome) in a sample of older adults. Since significant sex- and age-related differences were observed, these findings might serve in both clinical and large cohort studies to compare and generalize the level of CRF, especially in a ‘risky’ group of older adults. The data obtained in this study could be a potential start point for screening, monitoring and tracking cardiopulmonary functional status in older adults, with the practical implication of prognostic utility for some major non-communicable diseases. Also, sex- and age-related changes in VO_2peak_ values would be important for a worldwide comparison between different ethnic populations. By including cardiopulmonary exercise testing during systematic examinations, health-related professionals would be able to get a clearer picture of the overall health status of an individual and how these data may be implemented in everyday interventions.

## Figures and Tables

**Table 1:** Basic descriptive statistics of the study participants.

	Total Sample (N = 450)	Men (N = 170)	Women (N = 280)	*p*-Value
	Mean ± SD	Mean ± SD	Mean ± SD	
Age (years)	67.4 ± 5.4	67.8 ± 5.7	67.2 ± 5.2	0.295
Height (cm)	165.7 ± 8.6	173.5 ± 6.2	160.9 ± 6.0	<0.001
Weight (kg)	75.1 ± 13.3	83.3 ± 10.7	70.0 ± 12.2	<0.001
BMI (kg/m^2^)	27.1 ± 3.9	27.4 ± 3.4	26.9 ± 4.2	0.201
WC (cm)	93.9 ± 11.8	99.4 ± 9.7	90.6 ± 11.7	<0.001
WHR	0.57 ± 0.07	0.57 ± 0.06	0.56 ± 0.07	0.116
Fat mass (%)	34.5 ± 7.6	30.8 ± 6.9	36.7 ± 7.2	<0.001
SBP_resting_ (mmHg)	142.6 ± 18.8	145.2 ± 17.1	141.0 ± 19.6	0.024
DBP_resting_ (mmHg)	87.5 ± 10.4	87.8 ± 10.1	87.2 ± 10.6	0.555
HR_resting_ (bpm)	67.3 ± 9.9	64.9 ± 10.2	68.8 ± 9.4	<0.001
HR_max_ (bpm)	160.8 ± 3.8	160.6 ± 4.0	161.0 ± 3.6	0.295
Glucose (mmol/L)	5.8 ± 1.0	6.0 ± 1.4	5.6 ± 0.8	<0.001
Total cholesterol (mmol/L)	5.5 ± 1.3	5.0 ± 1.2	5.7 ± 1.2	<0.001

Abbreviations: BMI—body mass index; WC—waist circumference; WHR—waist-to-height ratio; SBP—systolic blood pressure; DBP—diastolic blood pressure; HR—heart rate; bpm—beats per minute; *p* < 0.05.

**Table 2:** Reference data for VO_2peak_ by sex and age (N = 450).

Sex	Age	P5	P10	P25	P50 (M)	P75	P90	P95
Men	60–64 years	26.3	29.5	33.1	36.1	40.7	45.3	51
	65–69 years	18.6	20.0	26.2	29.9	34.5	39.2	45.1
	70–74 years	16.3	18.7	20.5	23.7	28.4	33.6	35.5
	≥75 years	15.0	16.2	16.7	20.3	25.4	31.1	33.2
	Total	18.1	19.0	24.1	32.1	36.0	41.9	45.0
Women	60–64 years	22.4	25.5	27.1	29.7	33.0	35.1	40.8
	65–69 years	18.3	19.3	23.7	26.0	30.2	33.9	37.5
	70–74 years	15.7	17.1	19.8	21.0	25.5	28.3	31.2
	≥75 years	10.2	13.9	15.1	18.1	22.5	25.6	30.1
	Total	15.1	17.2	22.4	26.6	30.1	32.9	35.2

Abbreviations: P—percentile; M—median.

## Data Availability

The datasets used and/or analyzed during the current study are available from the corresponding author on reasonable request.
